# Supplementary material for: Retrospective Evaluation of Various Serological Assays and Multiple Parameters for Optimal Diagnosis of Lyme Neuroborreliosis in a Routine Clinical Setting
Source: Microbiol Spectr. 2022 Jun 21;10(3):e00061-22. doi: 10.1128/spectrum.00061-22 (PMC9241602; doi:10.1128/spectrum.00061-22)
Supplement: SUPPLEMENTAL FILE 1 — Supplemental material. Download spectrum.00061-22-s0001.pdf, PDF file, 0.8 MB [file spectrum.00061-22-s0001.pdf]

Retrospective Evaluation of Various Serological Assays and Multiple Parameters for Optimal Diagnosis of Lyme Neuroborreliosis in a Routine Clinical Setting

Authors:

T. van Gorkom,[a,c] W. Voet,[b] G.H.J. van Arkel,[a,c] M. Heron,[a] B.J.A. Hoeve-Bakker,[c] S.F.T. Thijsen,[a] K. Kremer[a]*

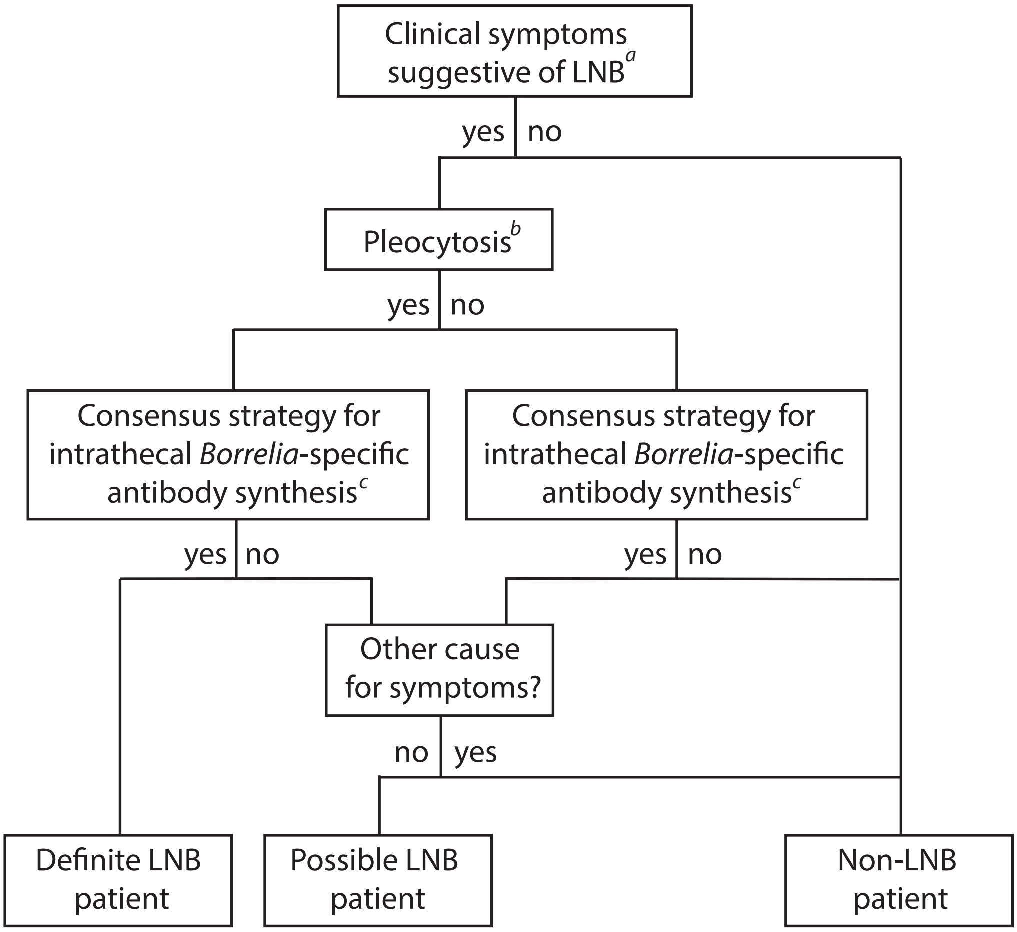

Supplemental figure S1. Flow chart used to classify the 156 patients included in this study into definite and possible Lyme neuroborreliosis (LNB) and non-LNB patients based on the criteria defined by the European Federation of Neurological Societies (EFNS) (1) and consensus strategy for proof of intrathecal *Borrelia*-specific antibody synthesis.

a. Clinical symptoms suggestive of LNB were assumed to be present when a request for the detection of intrathecal *Borrelia*-specific antibody synthesis was done at our laboratory at the time of active disease in the past for which the IDEIA LNB IgM and IgG assay (Oxoid, Hampshire, United Kingdom) was used.
b. Pleocytosis was based on a CSF leucocyte count ≥5 leucocytes/µl.
c. The consensus strategy entailed that intrathecal *Borrelia*-specific antibody synthesis was only considered proven if the majority of the CSF-serum assays under investigation (i.e. IDEIA, Medac ELISA, *recom*Bead assay, Serion ELISA and Enzygnost ELISA) showed a pathological *Borrelia*-specific IgM and/or IgG antibody index value (≥1.5).

Reference:

1. Mygland A, Ljostad U, Fingerle V, Rupprecht T, Schmutzhard E, Steiner I, European Federation of Neurological Societies. 2010. EFNS guidelines on the diagnosis and management of European Lyme neuroborreliosis. Eur J Neurol 17:8–16. https://doi.org/10.1111/j.1468-1331.2009.02862.x.

Supplemental table S2. The criteria for classification and the laboratory test results of the 156 patients included in this study, by study group (i.e. definite Lyme neuroborreliosis [LNB], possible LNB and non-LNB patients). Classification was done based on the criteria of the European Federation of Neurological Societies (EFNS (1)) and consensus strategy and comprised the presence of clinical symptoms suggestive of LNB without another cause, pleocytosis and intrathecal *Borrelia*-specific antibody synthesis (see also Figure S1). Intrathecal *Borrelia*-specific antibody synthesis was based on the consensus strategy, which entailed that intrathecal *Borrelia*-specific antibody synthesis was only considered proven if the majority of the cerebrospinal fluid (CSF)-serum assays under investigation showed a pathological *Borrelia*-specific IgM and/or IgG antibody index value (≥1.5). Within each study group, a row represents a unique combination of EFNS criteria and laboratory test results. Black boxes indicate the presence of the EFNS criterium or a positive laboratory test result, white boxes indicate the absence of the EFNS criterium or a negative laboratory test result. Within these boxes, M stands for an IgM response, G for an IgG response, and MG for an overall Ig (IgM and IgG) response. Grey boxes indicate missing values (ND, not determined).

		EFNS criteria (1)			Intrathecal *Borrelia*-specific antibody synthesis: CSF-serum assays					Intrathecal *Borrelia*-specific antibody synthesis: CSF-only assays		Two-tier serology on serum (a)										
Study group	No. of patients (b)	Presence of clinical symptoms suggestive of LNB (c)	Pleocytosis (≥5 leucocytes/µl)	Consensus strategy (d)	IDEIA	Medac ELISA	*recom*Bead assay	Serion ELISA	Enzygnost ELISA	C6 ELISA	*recom*Line immunoblot (e)	C6 ELISA	*recom*Line immunoblot (e)	*Borrelia* species PCR on CSF	CXCL13 result on CSF	Elevated total protein in CSF	Dysfunctional blood-CSF barrier	Intrathecal total Ab synthesis (>10%) (f)	Reibergram classification (g)	Clinical symptoms/ diagnosis (h)	Symptom duration (i)	Antibiotic treatment for LNB (j)
**Definite LNB**																						
n=10	1	■	■	5/5	MG	G	G	MG	MG	■	G	■	MG	■	■	■	■	MG	area 3	Radiculopathy and erythema migrans	43	ctrx 28
	1	■	■	5/5	G	G	MG	MG	MG	■	G	■	G		■	■	■	MG	area 3	Radiculopathy	108	ctrx 14
	1[a]	■	■	4/4	G	G	G	G	ND[n]	■	G	■	G		■		■		area 2	Radiculopathy and cognitive impairment	63	ctrx 28
	1[a]	■	■	4/4	G	MG	MG	G	ND[n]	■	G	■	MG		■	■	■	MG	area 3	Myelitis transversa	3	ctrx 14
	1[a]	■	■	4/4	MG	MG	MG	MG	ND[n]	■	G	■	G	■	■	■	■	MG	area 3	Radiculopathy and cognitive impairment	32	ctrx 14
	1	■	■	4/4	G	G	G	G	ND[n]	■	G	■	G		■				area 1	Radiculopathy	14	ctrx 14
	1[a]	■	■	4/4	G	G	G	MG	ND[n]	■	G[o]	■	G		■	■	■	MG	area 3	Polyradiculopathy with cranial (nerve VII) and peripheral neuropathy	21	ctrx 28
	1	■	■	3/4		G	MG	ND[m]	G	■	G	■	G		■	■	■	M	area 3	Radiculopathy	174	ctrx/doxy
	1	■	■	4/5		MG	G	MG	MG	■		■	MG		■		■	M	area 3	Cranial neuropathy (nerve VII)	22	ctrx 14
	1	■	■	4/5		MG	G	G	G	■		■					■		area 2	Cranial neuropathy (nerve VII) and tickbite	5	ctrx 14
Total	10	10	10	10	7	10	10	9	5	10	8[o]	10	9	2	9	6	9	7	area 1: n=1<br>area 2: n=2<br>area 3: n=7<br>area 4: n=0			
**Possible LNB**																						
n=4	1	■	■	2/5		M			M	■		■			■		■	M	area 3	Cranial neuropathy (nerve V)	47	ctrx 28
	1	■	■	1/4			ND[k]		M	■		■	MG						area 1	Cranial neuropathy (nerve VII)	13	ctrx 14
	1[a]	■	■	0/4					ND[n]	■		■	MG				■		area 2	Cranial neuropathy (nerve VII)	8	ctrx 14
	1[a]	■	■	0/3			ND[l]		ND[n]			■	G						area 1	Cranial neuropathy (nerve VI)	39	ctrx 14
n=3	1	■		5/5	MG	G	G	G	G	■		■	G					M	area 4	Peripheral neuropathy	288	doxy 30
	1	■		4/5		G	G	G	G	■	G[o]	■	G				■		area 2	Peroneus neuropathy	79	
	1	■		4/5		G	G	MG	G	■		■	MG					M	area 4	White matter lesions	229	
Total	7	7	4	3	1	4	3	3	5	6	1[o]	7	6	0	1	0	3	3	area 1: n=2<br>area 2: n=2<br>area 3: n=1<br>area 4: n=2			

non-LNB

n=3	1	■	■	1/5					G						■	■	■	MG	area 3	Neurosyphilis
	1	■	■	0/4			ND[l]			■	G[o]	■	G						area 1	Peripheral neuropathy (isolated paralysis flexor pollicis due to a Schwannoma in shoulder)
	1	■	■	0/5								■	G						area 1	Previously treated LNB (76 days prior)
n=36	1	■		2/5			G		M	■	G[o]	■	G						area 1	General malaise, black outs, no final diagnosis made
	1	■		1/5			G			■	G[o]	■	G						area 1	Acrodermatitis chronica atrophicans
	2	■		1/5			G					■	G						area 1	Nonspecific tendon-myogenic pain (n=1); Peripheral neuropathy (n=1)
	1	■		2/5				G	G			■	G[o]			■			area 1	Demyelinating condition
	2	■		1/5				G				■	G						area 1	Peripheral neuropathy (tricheminus neuralgie) (n=1); Spinal stenosis (n=1)
	1	■		1/5					G			■	M					M	area 4	Demyelinating condition (MS)
	1	■		0/5						■	G[o]	■	G				■		area 2	
	1	■		0/5						■		■	G						area 1	
	1	■		0/5								■	G			■	■		area 2	
	1	■		0/5							G[o]	■	G				■		area 2	
	1	■		0/5								■	M						area 1	
	12	■		0/5								■	G						area 1	
	3	■		0/5								■	MG						area 1	
	1	■		0/5								■					■		area 2	
	1	■		0/5								■	G[o]						area 1	
	1	■		0/5															area 1	
	5	■		0/5								■							area 1	
n=19	1		■	0/4				ND[m]							■	■	■		area 2	Tuberculous meningitis
	1		■	0/5													■		area 2	
	1		■	0/5									M[p]				■		area 2	
	3		■	0/5												■	■		area 2	
	1		■	0/5														M	area 4	
	3		■	0/5														G	area 4	
	1		■	0/5														MG	area 4	
	8		■	0/5															area 1	
n=81	1			1/5				G										G	area 4	Treated neurosyphilis
	2			1/5				G											area 1	Panic disorder (n=1); Radiculopathy (n=1)
	1			0/5									M[p]				■		area 2	
	7			0/5													■		area 2	
	3			0/5												■	■		area 2	
	3			0/5														M	area 4	
	3			0/5														G	area 4	
	1			0/5								■							area 1	
	2			0/5									M[p]						area 1	
	58			0/5															area 1	
Total	139	39	22	0	0	0	4	6	4	5	5[o]	38	31[o] (+4[p])	0	2	10	22	14	area 1: n=104<br>area 2: n=21<br>area 3: n=1<br>area 4: n=13	
**Total (all patients)**	156	56	36	13	8	14	17	18	14	21	14[o]	55	46[o] (+4[p])	2	12	16	34	24	area 1: n=107<br>area 2: n=25<br>area 3: n=9<br>area 4: n=15	

a. Two-tier serology on serum was performed using the C6 ELISA as a screening test, and positive (and equivocal) C6 ELISA results were confirmed using the *recom* Line IgM and IgG immunoblot.

b. One hundred and fifty (96.2%) of the 156 study participants were consecutive patients who were eligible for inclusion if a CSF and a blood sample (drawn within 24 h of the lumbar puncture) had been sent to the microbiology laboratory of the Diakonessenhuis Hospital, Utrecht, the Netherlands, in the period between August 2013 and June 2016. Six (3.8%) of the 156 study participants (four definite and two possible LNB patients; all of them are marked with 'a' in the table) were selected from outside this period (from February 2011 to July 2013 and from July 2016 to November 2017).

c. Clinical symptoms suggestive of LNB were assumed to be present when a request for LNB diagnostics was done at the time of active disease in the past, which included the detection of intrathecally-produced *Borrelia* -specific IgM and IgG using the IDEIA.

d. The consensus strategy entailed that intrathecal *Borrelia* -specific antibody synthesis was only considered proven if the majority of the CSF-serum assays under investigation (i.e. IDEIA, Medac ELISA, *recom* Bead assay, Serion ELISA and Enzygnost ELISA) showed a pathological *Borrelia* -specific IgM and/or IgG antibody index value (≥1.5).

e. The IgG results are based on the revised interpretation criteria of the *recom* Line IgG immunoblot implemented by the manufacturer in January 2019 (Table S3).

f. Intrathecal total antibody (Ab) synthesis is proven if the intrathecal total IgM and/or total IgG fraction is larger than 10% as described by Reiber (2).

g. The Reibergram classification is based on the blood-CSF barrier functionality and the presence of intrathecal total antibody (IgM and/or IgG) synthesis (2). The four areas listed in this column are explained as follows: area 1, a normal blood-CSF barrier without intrathecal total antibody synthesis; area 2, a dysfunctional blood-CSF barrier without intrathecal total antibody synthesis; area 3, a dysfunctional blood-CSF barrier with intrathecal total antibody synthesis; and area 4, a normal blood-CSF barrier with intrathecal total antibody synthesis, the results of which have been published previously (3).

h. Clinical symptoms for patients who had a pathological IgM and/or IgG AI value in at least one of the five CSF-serum assays, and/or a positive test result in at least one of the two CSF-only assays, and/or a positive *Borrelia* species PCR result on CSF, and/or a positive CSF-CXCL13 result are listed in the corresponding row and if this included patients for whom LNB was ruled out, the alternative diagnosis was shown. For all other (non-LNB) patients, diagnoses that were found at least twice included peripheral neuropathy (n=18), demyelinating conditions (n=15) including six cases of multiple sclerosis (MS), radiculopathy (n=8), non-CSF infectious disease (n=7), spinal stenosis (n=6), (transient) facial nerve paralysis (n=6), proven non-LNB CSF infectious disease (n=3; streptococcus (n=1) and viral (n=2) meningitis); nonspecific tendon-myogenic pain (n=3), cerebrovascular accident (n=3), headache/migraine (n=3), cancer (n=3), epilepsy (n=2), sleep disorder (n=2), psychogenic disorder (n=2), microvascular white matter lesions (n=2), and arthralgia (n=2). A unique diagnosis was found for 18 non-LNB patients and 21 non-LNB patients never received a diagnosis (data not shown).

i. Symptom duration is the number of days between the start of symptoms and the lumbar puncture and is only shown for definite and possible LNB patients.

j. Of the 17 patients that were classified as definite or possible LNB patient, 15 (10 definite and 5 possible LNB patients) had been treated for LNB following the recommendations of the Dutch guidelines for LB (4). This treatment had started after the LP was performed with a median of 0 days [range 0-18] after CSF-blood sampling. Nine of the 10 definite LNB patients were treated with ceftriaxone (2 g/day) intravenously for either 14 (n=6; [ctrx 14]) or 28 (n = 3; [ctrx 28]) days. One definite LNB patient had started with intravenous ceftriaxone (2 g/day), but after 5 days switched to oral doxycycline (100 mg twice a day) for 25 days because of an allergic reaction (ctrx/doxy). Four of the five possible LNB patients received ceftriaxone (2 g/day) intravenously for either 14 (n=3; [ctrx 14]) or 28 (n = 1; [ctrx 28]) days. The remaining possible-LNB patient received oral doxycycline (100 mg twice a day) from the start for 30 days (doxy 30).

k. For one possible LNB patient, the IgM antibody index value using the *recom* Bead assay is missing due to insufficient sample material (for this patient, the IgG antibody index value using the *recom* Bead assay is normal).

l. For two cases, one possible LNB and one non-LNB patient, the IgG antibody index value using the *recom* Bead assay is missing due to insufficient sample material (for these patients, the IgM antibody index value using the *recom* Bead assay is normal).

m. For two cases, one definite LNB and one non-LNB patient, the IgM and IgG antibody index values using the Serion ELISA were not determined due to insufficient sample material.

n. For seven cases, five definite and two possible LNB patients, the IgM and IgG antibody index values using the Enzygnost ELISA were not determined as the ELISA was taken of the market.

o. The positive overall Ig *recom* Line immunoblot result was based on a negative result for IgM and a positive result for IgG. For IgG, this result was based on the revised interpretation criteria of the *recom* Line IgG immunoblot (Table S3); however, using the old interpretation criteria, the IgG result on CSF for seven cases (one definite LNB, one possible LNB and five non-LNB patients) and the IgG result on serum for two non-LNB patients, was negative. Consequently, the overall Ig result based on the old interpretation criteria of the *recom* Line IgG immunoblot would also have been negative for these cases.

p. The *recom* Line immunoblot was tested on all 156 sera and the results were compared with those obtained in CSF to assess the origin of the *Borrelia* -specific antibodies (CSF or blood-derived). For four non-LNB patients, all of whom had a negative C6 ELISA result on serum and were, thus, considered seronegative using the two-tier testing algorithm, the *recom* Line IgM immunoblot on serum was positive. For three of them, IgM *recom* Line immunoblot positivity was based on a positive band for either one (n=2) or three (n=1) OspC antigens. For one non-LNB patient, IgM *recom* Line immunoblot positivity was based on a positive band for p41 and p18 *B. garinii* .

References:

1. Mygland A, Ljostad U, Fingerle V, Rupprecht T, Schmutzhard E, Steiner I, European Federation of Neurological Societies. 2010. EFNS guidelines on the diagnosis and management of European Lyme neuroborreliosis. Eur J Neurol 17:8–16.
2. Reiber H. 1995. External quality assessment in clinical neurochemistry: survey of analysis for cerebrospinal fluid (CSF) proteins based on CSF/serum quotients. Clin Chem41:256–263. https://doi.org/10.1093/clinchem/41.2.256.
3. van Gorkom T, van Arkel GHJ, Heron M, Voet W, Thijsen SFT, Kremer K. 2021. The usefulness of two CXCL13 assays on cerebrospinal fluid for the diagnosis of Lyme neuroborreliosis: a retrospective study in a routine clinical setting. J Clin Microbiol 59:e0025521. https://doi.org/10.1128/JCM.00255-21.
4. Kwaliteitsinstituut voor de Gezondheidszorg (CBO). 2013. Dutch guidelines Lyme disease 2013. CBO, The Netherlands. http://www.tekenbeetziekten.nl/wp-content/uploads/2014/08/CBO-richtlijn-Lymeziekte-versie-2013.pdf.

Supplemental table S3. Test characteristics of the five antibody assays tested on CSF-serum pairs (A) and the two antibody assays tested on CSF only (B) used for the detection of intrathecally produced *Borrelia*-specific antibodies.

Assays	Technique	Target	Quantification	Intrathecal antibody synthesis	Normal AI value	Borderline AI value	Pathological AI value
**A.**							
IDEIA[TM] Lyme Neuroborreliosis	Capture ELISA	IgM and IgG: purified native DK1 flagellum (p41) *B. afzelii*	Semi-quantitative; OD	AI by using the formula: (OD CSF/ OD SER) * (OD CSF - OD SER)	AI < 0.3 or OD CSF <0.150	N/A	AI ≥ 0.3
Borrelia-IgM-ELISA Medac<br>Borrelia-IgG-ELISA Medac	ELISA	IgM: OspC and VlsE<br>IgG: VlsE	Quantitative (concentration using single-point quantification with lot-specific calibration curve)	AI by Reiber and Peter (4)	0.6 ≤ AI ≤ 1.3	1.3 < AI ≤ 1.5[f]	AI > 1.5
*recom*Bead Borrelia IgM 2.0<br>*recom*Bead Borrelia IgG 2.0	Luminex	IgM and IgG against highly purified recombinant antigens from *Bbss, Bafz, Bgar, Bbav,* and *Bsp*. Different fluorescent-labelled beads are coated with either one of the following antigens: p100, VlsE, p58, p39, OspA, OspC (*Bbss, Bafz, Bgar*)[a], and p18 (*Bbss, Bafz, Bbav, Bgar, Bsp*)[a,b]	Semi-quantitative; MFI	For each antigen separately, an AI is calculated by Reiber and Peter (4). A single pathological AI value is sufficient to suggest the intrathecal synthesis of *Borrelia*-specific antibodies	0.6 ≤ AI ≤ 1.3	1.3 < AI < 1.5[f]	AI ≥ 1.5
Borrelia burgdorferi IgM SERION ELISA classic<br>Borrelia burgdorferi IgG SERION ELISA classic	ELISA	IgM: whole cell lysates *B. afzelii* Pko (1) and *B. garinii* (2)<br>IgG: whole cell lysates *B. afzelii Pko* (1) *and B. garinii* (2) + recombinant VlsE	Quantitative (concentration using single-point calibration with 4-PL method and lot-specific calibration curve)	AI by Reiber and Peter (4)	0.7 ≤ AI ≤ 1.4	N/A	AI > 1.5
Enzygnost® Borreliosis/IgM<br>Enzygnost® Lyme link VlsE/IgG	ELISA	IgM: whole cell lysate *B. burgdorferi* PKo<br>IgG: whole cell lysate *B. afzelii* PKo + recombinant VlsE	Quantitative (OD index for IgM and concentration using the alfa-method for IgG)	AI by Reiber and Peter (4)	0.5 ≤ AI ≤ 1.49	N/A	AI ≥ 1.5
**B.**							
C6 ELISA	ELISA	Total Ig (IgM and IgG) against synthetic C6 peptide, derived from a highly immunogenic part (invariable region 6) of the VlsE lipoprotein (3)	Semi-quantitative by Lyme Index[c]	N/A	N/A	N/A	N/A
*recom*Line IgM and IgG (5,6)[d]	Immunoblot	IgM and IgG against various antigens from *Bbss, Bafz, Bgar, Bbav,* and *Bsp.* Antigens coated are p100, VlsE, p58, p41, p39, OspA, OspC (*Bbss, Bafz, Bgar, Bsp*)[a], and p18 (*Bbss, Bafz, Bbav, Bgar2, Bsp*)[a,b]	Qualitative by intensity and number of bands[d,e]	N/A	N/A	N/A	N/A

AI, antibody index; Ig, immunoglobulin; OD, optical density; CSF, cerebrospinal fluid; SER, serum; N/A, not applicable; Osp, outer surface protein; VlsE, variable major protein-like sequence, expressed; Bbss, *B. burgdorferi sensu strictu*; Bafz, *B. afzelii*, Bgar, *B. garinii*; Bbav, *B. bavariensis*; Bsp, *B. spielmanii*; MFI, median fluorescence intensity; 4-PL, 4-parameter logistic

a. Reactivity against multiple OspC antigens is counted only once, irrespective of how many OspC antigens react. This also applies for reactivity against multiple p18 antigens.

b. p18 = DbpA, decorin binding protein.

c. For both CSF and serum: C6 ELISA results were interpreted by the calculation of a Lyme index (LI) for which the following formula was used: LI = OD sample / (OD calibrator + 0.3) as described in the instruction manual of the manufacturer for serum. Subsequently, results were classified as either negative (LI ≤ 0.90), equivocal (0.91 < LI < 1.09), or positive (LI ≥ 1.10).

d. The manufacturer of the *recom*Line immunoblot revised the interpretation of the *recom*Line IgG immunoblot in January 2019 by increasing the point value of the VlsE band from 5 to 6, which has an effect on the test result. In the current study, both the old and the revised interpretation criteria were used; however, only the revised interpretation criteria were elaborated on in the manuscript.

e. For both CSF and serum: point values for IgM-bands: p100 = 5, VlsE = 5, p58 = 4, p41 (flagellin) = 1, p39 = 4, OspA = 5, OspC = 8, p18 (DbpA) = 5. Point values for IgG-bands: p100 = 5, VlsE = 5, p58 = 4, p41 (flagellin) = 1, p39 = 5, OspA = 5, OspC = 5, p18 (DbpA) = 5. Results are based on the sum of the point values of the bands that have an equal or larger intensity than the cutoff band and are interpreted as negative (≤5 points), equivocal (6 points), or positive (≥7 points).

f. AI values that were equivocal should have been repeated; however, this was not done due to limited sample material.

References

1. Canica MM, Nato F, du ML, Mazie JC, Baranton G, Postic D. 1993. Monoclonal antibodies for identification of *Borrelia afzelii* sp. nov. associated with late cutaneous manifestations of Lyme borreliosis. Scand J Infect Dis 25:441-448. https://doi.org/10.3109/00365549309008525

2. Baranton G, Postic D, Saint G, I, Boerlin P, Piffaretti JC, Assous M, Grimont PA. 1992. Delineation of *Borrelia burgdorferi sensu stricto*, *Borrelia garinii* sp. nov., and group VS461 associated with Lyme borreliosis. Int J Syst Bacteriol 42:378-383. https://doi.org/10.1099/00207713-42-3-378

3. Liang FT, Steere AC, Marques AR, Johnson BJ, Miller JN, Philipp MT. 1999. Sensitive and specific serodiagnosis of Lyme disease by enzyme-linked immunosorbent assay with a peptide based on an immunodominant conserved region of *Borrelia burgdorferi* VlsE. J Clin Microbiol 37:3990–3996. https://doi.org/10.1128/JCM.37.12.3990-3996.1999.

4. Reiber H, Peter JB. 2001. Cerebrospinal fluid analysis: disease-related data patterns and evaluation programs. J Neurol Sci 184:101–122. https://doi.org/10.1016/s0022-510x(00)00501-3.

5. Mikrogen Diagnostik GmbH, Neuried, Germany. Instruction manual *recom*Line IgM and IgG. July 2013.

6. Mikrogen Diagnostik GmbH, Neuried, Germany. Instruction manual *recom*Line IgM and IgG. January 2019.
